# Detectable depth of unexposed parathyroid glands using near-infrared autofluorescence imaging in thyroid surgery

**DOI:** 10.3389/fendo.2023.1170751

**Published:** 2023-04-11

**Authors:** Young Seok Han, Yikeun Kim, Hyoung Shin Lee, Yeongjoon Kim, Yeh-Chan Ahn, Kang Dae Lee

**Affiliations:** ^1^ Department of Otorhinolaryngology-Head and Neck Surgery, Kosin University Gospel Hospital, Kosin University College of Medicine, Busan, Republic of Korea; ^2^ Department of Biomedical Engineering, Ulsan National Institute of Science and Technology (UNIST), Ulsan, Republic of Korea; ^3^ Department of Biomedical Engineering and Industry 4.0 Convergence Bionics Engineering, Pukyong National University, Busan, Republic of Korea

**Keywords:** near-infrared autofluorescence, parathyroid gland, mapping, depth, thyroidectomy

## Abstract

**Background:**

Near-infrared light can penetrate the fat or connective tissues overlying the parathyroid gland (PG), enabling early localization of the PG by near-infrared autofluorescence (NIRAF) imaging. However, the depth at which the PG can be detected has not been reported. In this study, we investigated the detectable depth of unexposed PGs using NIRAF during thyroidectomy.

**Materials and methods:**

Fifty-one unexposed PGs from 30 consecutive thyroidectomy patients, mapped by an experienced surgeon (K.D. Lee) with the use of NIRAF imaging, were included. For NIRAF detection of PGs, a lab-built camera imaging system was used. Detectable depths of the unexposed PGs were measured using a Vernier caliper. The NIRAF images were classified as faint or bright depending on whether a novice could successfully interpret the image as showing the PG. Data on variables that may affect detectable depth and NIRAF intensity were collected.

**Results:**

Detectable depth ranged between 0.35 and 3.05 mm, with a mean of 1.23 ± 0.73 mm. The average NIRAF intensity of unexposed PGs was 3.13 au. After dissection of the overlying tissue, the intensity of the exposed PG increased to 4.88 au (p < 0.001). No difference in NIRAF intensity between fat-covered (3.27 ± 0.90 au) and connective tissue-covered PGs (3.00 ± 1.23 au) was observed (p = 0.369). PGs covered by fat tissue (depth: 1.77 ± 0.67 mm) were found at deeper locations than those covered by connective tissue (depth: 0.70 ± 0.21 mm) (p < 0.001). The brightness of images of the faint group (2.14 ± 0.48 au) was on average 1.24 au lower than that of the bright group (3.38 ± 1.04 au) (p = 0.001). A novice successfully localized 80.4% of the unexposed PGs. Other variables did not significantly affect detectable depth.

**Conclusion:**

Unexposed PGs could be mapped using NIRAF imaging at a maximum depth of 3.05 mm and an average depth of 1.23 mm. A novice was able to localize the PGs before they were visible to the naked eye at a high rate. These results can be used as reference data for localization of unexposed PGs in thyroid surgery.

## Introduction

Preservation of a healthy parathyroid gland (PG) is a key element of successful thyroidectomy. Postoperative loss of function may lead to temporary or permanent hypoparathyroidism or hypocalcemia. These conditions are often asymptomatic, but in some cases cause symptoms such as paresthesia, rigidity, arrhythmias, seizures, and even death (1).

Since healthy PGs are usually small in size and similar in color and shape to the surrounding adipose tissue or lymph nodes, there is always a risk of inadvertent resection of PGs, especially by novice surgeons (2). Paek et al. report that inexperience of the surgeon is a significant risk factor for permanent hypoparathyroidism. They demonstrated that permanent hypoparathyroidism occurs in 6.5% of patients treated by a surgeon in the first 2 years of practice, decreasing to 1.8% in the case of a surgeon in the next 2 years (3). Furthermore, a report has shown that, even when experienced surgeons believe that the PGs have been well preserved during thyroidectomy, the PGs have in fact been inadvertently removed in up to 22% of cases (4). As a solution to these problems, near-infrared autofluorescence (NIRAF) imaging, which can improve identification and preservation of PGs during thyroidectomy, has recently emerged (5). With sensitivity and accuracy ranging from 90% to 100% (6–10), NIRAF imaging can identify the PGs in 90% to 100% of cases (6–8, 11–13).

In addition, as NIR light can penetrate human tissues up to 4.0 mm deeper than visible light, unexposed PGs can be detected before they are visible to the naked eye via NIRAF imaging (14). The process of identifying PGs that are invisible to the naked eye using NIRAF imaging is referred to as “PG mapping” (10, 15). It is reported that 37–92% of unexposed PGs can be localized using this mapping procedure (6, 10). If the imaging technique can detect the PGs more readily than the surgeon’s naked eye during routine thyroidectomy, it may offer a greater chance of avoiding damage to healthy PGs. This may be of practical use in minimizing cases of hypoparathyroidism. However, there have been no reports on the range of depths at which the autofluorescence emitted from the biological fluorophore of the PGs can be detected by NIRAF imaging. In this study, we investigated the detectable depth of unexposed PGs under NIRAF imaging and the brightness of NIRAF intensity required for a novice to interpret the images as PGs during thyroidectomy.

## Materials and methods

This prospective study was approved by the Institutional Review Board (IRB) of Kosin University Gospel Hospital (IRB No. 2021-07-008-001). Informed consent was obtained from all the participants at the beginning of the study.

### Patient selection

Thirty consecutive patients (5 men, 25 women; mean age, 52.1 ± 9.5 years; range, 36.0–67.0 years) requiring thyroidectomy (26 cases of papillary thyroid cancer, 1 case of medullary thyroid cancer, 3 cases of follicular adenoma) were enrolled in the study at Kosin University Gospel Hospital between August and October 2021. Patients diagnosed with malignant tumors underwent elective or therapeutic central neck dissection (CND). A total of 78 PGs were expected to be found in nine total thyroidectomies and 21 lobectomies.

### Data collection

The background clinical variables measured included sex (male or female); age (dichotomized as above or below the mean: <52 or ≥52 years); body mass index (BMI) at the time of surgery (categorized as normal (18.5–23 kg/m^2^) or overweight or obese (≥23 kg/m^2^)); and preoperative levels of serum parathyroid hormone (PTH, categorized as normal (10–65 pg/mL) or high (>65 pg/mL)), serum calcium (categorized as normal (8.5–10.5 mg/dL) or high (>10.5 mg/dL)), and total 25-hydroxyvitamin D (vitamin D, categorized as low (<20 ng/mL) or normal (20–100 ng/mL)).

Several other variables were also measured: depth from the superficial surface of the overlying tissue to the superficial surface of the PG; type of overlying tissue (connective or fat tissue); location of the PG (superior or inferior); NIRAF intensity as a ratio of parathyroid to background intensity (P/B ratio); and postoperative final diagnosis (malignant, benign, or thyroiditis). P/B ratio was calculated using ImageJ software with NIRAF images captured at the moment when fluorescence was most evident. NIRAF intensity is expressed in arbitrary units (au). A P/B ratio greater than 1 indicates that the PG is brighter than the surrounding tissues.

In order to determine whether there was a difference between novice and expert interpretation of NIRAF images, all images were evaluated by both a novice and an expert. The images were classified into two groups (faint or bright) based on the novice’s evaluation. Specifically, the novice assessed NIRAF video images that were recorded during the expert’s PG mapping process. The images covered the entire field from the thyroid superior pole to the inferior pole and the thymic tongue area. The novice reviewed these images independently after the surgical procedure without any guidance from or annotations by the expert. There were no clues in the NIRAF video images that could suggest the presence of PGs. The novice (Y.S. Han) was a second-year Otolaryngology–Head & Neck Surgery resident who joined in the first year of thyroidectomy as an assistant at the beginning of the study and had no prior experience with NIRAF imaging. The expert (K.D. Lee) had over 25 years of experience, had handled >5,000 cases of thyroidectomy as of the beginning of the study, and had over 7 years of experience with NIRAF imaging. Among the images that the expert was convinced of as showing PGs, those images in which the inexperienced resident could not be sure of the presence of PGs were defined as “faint images”. Those images in which the resident was also confident of the autofluorescence were defined as “bright images”.

### Equipment for NIRAF imaging and depth measurement

**Figure 1:**
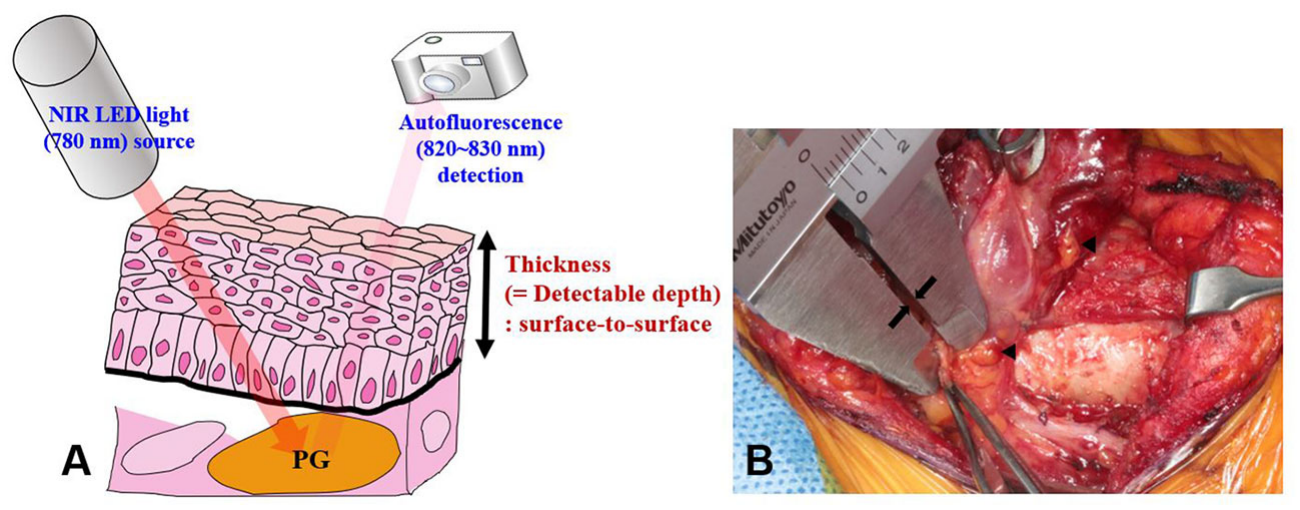
Measurement of the detectable depth of the PG. **(A)** When the PG is illuminated with 780 nm NIR light, it emits autofluorescence of wavelength 820–830 nm. The camera detects the emitted light. **(B)** Detectable depth was measured by a highly experienced surgeon (K.D. Lee) using a Vernier caliper (see arrows). Arrowheads indicate PGs. NIRAF, near-infrared autofluorescence; PGs, parathyroid glands.

The equipment used for NIRAF imaging in this study was developed by our research team and approved for research purposes by the Korean Ministry of Food and Drug Safety (Approval No. 1193). The NIRAF imaging device consisted of a digital single-lens reflex camera (Canon EOS Rebel T3, Tokyo, Japan) and a light-emitting diode light source (THORLABS M780L3-C1, Santa Barbara, CA, USA). A filter with a wavelength of 780 nm was applied to the light source to activate PGs in the target area. The camera was able to detect autofluorescence within the wavelength range of 820–830 nm (**Figure 1A**). The operating lights were turned off during NIRAF imaging to avoid interference with PG mapping. However, the fluorescent lamps in the operating room were kept on to aid surgical procedures and personnel movement. The light source intensity was the same (6 mW) during PG mapping for all patients. The distances of the digital single-lens reflex camera and NIR light-emitting diode light source from the target areas were kept constant at 85 ± 10 cm and 15 ± 5 cm, respectively, in all patients. The NIRAF imaging system, including the computer and display monitor, was the same equipment as described in previous studies (10, 16). Detectable depth was measured during surgery using a Vernier caliper (Mitutoyo, Kawasaki, Japan; accuracy of 0.05 mm) as the thickness of the tissue overlying unexposed PGs.

### Process of PG mapping and depth measurement

All surgeries were open thyroidectomies performed by a single surgeon (K.D. Lee). To minimize interruption to the surgical workflow, the time for PG mapping was set to less than 1 minute. If the PG was not located within this time, the surgery proceeded without further efforts to detect it. The entire time for NIRAF mapping and measurement of detected depth was set to less than five minutes.

NIRAF imaging of healthy PGs was performed in three stages: P1, P2, and P3. Stage P1 was defined as the stage at which PGs were not yet discernible to the surgeon’s eye and were only visible on NIRAF imaging. Once a PG was identified at stage P1, the first NIRAF image was recorded before further dissection. The area was marked with a marking pen, and its surface was meticulously dissected until the PG was exposed to the naked eye. The thickness of the overlying tissue was measured using a Vernier caliper from the surface of the dissected overlying tissue to the surface of the PG, avoiding tension or compression of the tissue as much as possible (**Figure 1**). All measurement procedures were performed by the experienced surgeon (K.D. Lee). Stage P2 was defined as the NIRAF imaging carried out at the stage after the surgeon’s visual identification of the PG. This second NIRAF image was recorded after dissection of the overlying tissue and depth measurement. When autofluorescence was not observed at stages P1 and P2, NIRAF imaging was carried on the excised surgical specimen; this was defined as stage P3.

We relied on the confidence level of the visual assessment of the experienced surgeon (K.D. Lee) to validate the accuracy of NIRAF imaging in order to avoid ethical issues and unnecessary injury to healthy PGs. Accuracy was defined as positive when the glowing area representing autofluorescence was consistent with the surgeon’s judgment of the presence of the PG. If the glowing area was not consistent with the surgeon’s judgment, accuracy was considered negative. Cases of inadvertently resected PG were defined as cases in which the PG was not identified intraoperatively but its presence was confirmed in histopathology.

Of 78 PGs, 51 images of unexposed PGs mapped by NIRAF imaging before identification by the naked eye were included in the study. The remaining 27 cases were excluded from the study for the following reasons: natural exposure to the surgeon’s naked eye (11 PGs); PG not identified during the surgical procedure (13 PGs); PG identified after dissection of the overlying tissue (1 PG, stage P2), false-positive NIRAF imaging (1 PG, stage P3); or PG detected in the extracted specimen (1 PG, stage P3).

### Statistical analysis

Statistical analysis was performed using IBM SPSS Statistics for Windows, version 28.0 (IBM Corp., Armonk, NY, USA). For continuous variables, a paired or independent t-test was used to compare means between groups. Categorical variables were analyzed using Pearson’s chi-squared test or Fisher’s exact test. All comparisons with a p-value of <0.05 were considered statistically significant.

## Results

Demographic and clinical data on 51 PGs were collected from 30 patients. Mean BMI was 23.79 ± 3.26 (18.93–30.05) kg/m^2^. Mean serum PTH, calcium, and vitamin D levels were 38.45 ± 13.80 (15.50–59.40) pg/mL, 9.6 ± 0.4 (9.0–10.5) mg/dL, and 27.17 ± 12.43 (8.50–64.95) ng/mL, respectively.

### Measured depth and NIRAF intensity of unexposed PGs

The maximum measured detectable depth 3.05 mm, the minimum was 0.35 mm, and the average was 1.23 mm (**Table 1**). The average NIRAF intensity was 3.13 au for unexposed PGs before dissection of the overlying tissue and 4.88 au after exposure (p < 0.001). The average depth was 0.70 mm in cases of overlying connective tissue and 1.77 mm in cases of overlying fat tissue (p < 0.001). In contrast, there were no significant differences in detectable depth between groups when participants were grouped on variables including sex, age, BMI, presence of thyroiditis, serum PTH, calcium, and vitamin D levels. Regarding the location of PGs, the average detected depths of the superior and inferior PGs were 1.42 and 1.04 mm, respectively (p = 0.061). No differences were found in NIRAF intensity according to location of the PG (p = 0.209) or overlying tissue type (p = 0.369) (**Table 1**). There was no significant difference in the distribution of tissue types (connective vs. fat) overlying superior versus inferior PGs (p = 0.886). Among the 26 PGs covered with connective tissue, 13 PGs (50%) were located superiorly and 13 (50%) inferiorly. Similarly, among the 25 PGs covered with fat tissue, 12 PGs (48%) were superior and 13 (52%) were inferior.

**Table 1:** Detectable depth and NIRAF intensity of unexposed PGs.

Variable	No. (%) of PGs; n = 51	Depth, mm		NIRAF intensity, au	
		Mean ± SD (range)	p	Mean ± SD (range)	p
Depth	51	1.23 ± 0.73 (0.35–3.05)			
					<0.001*
Before dissection	51			3.13 ± 1.07 (1.47–6.34)	
After dissection	51			4.88 ± 2.29 (2.06–11.47)	
Location of the PG			0.061		0.209
Superior	25 (49.0)	1.42 ± 0.79 (0.60–3.05)		3.33 ± 1.15 (1.60–6.34)	
Inferior	26 (51.0)	1.04 ± 0.62 (0.35–2.45)		2.95 ± 0.99 (1.47–6.03)	
Overlying tissue			<0.001*		0.369
Connective	26 (51.0)	0.70 ± 0.21 (0.35–1.15)		3.00 ± 1.23 (1.47–6.34)	
Fat	25 (49.0)	1.77 ± 0.67 (0.50–3.05)		3.27 ± 0.90 (1.83–6.03)	
Brightness			0.964		0.001*
Faint	10 (19.6)	1.24 ± 0.92 (0.35–2.80)		2.14 ± 0.48 (1.60–3.16)	
Bright	41 (80.4)	1.22 ± 0.69 (0.35–3.05)		3.38 ± 1.04 (1.47–6.34)	

*p <0.05. SD, standard deviation; NIRAF, near-infrared autofluorescence; PG, parathyroid gland.

### PG mapping using NIRAF imaging: Novice vs. experienced surgeon

**Figure 2:**
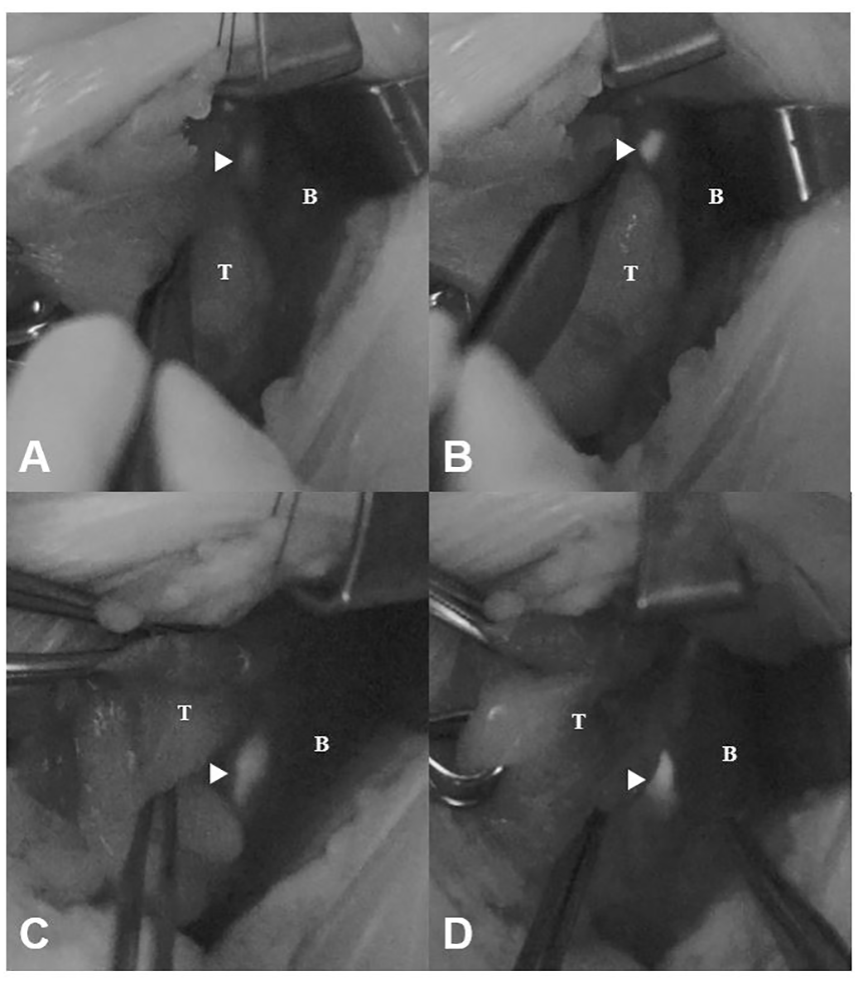
Faint and bright NIRAF images of unexposed and exposed PGs (arrowheads). **(A)** Faint image before dissection (NIRAF intensity: 2.15 au). **(B)** Faint image after dissection (NIRAF intensity: 4.01 au). **(C)** Bright image before dissection (NIRAF intensity: 3.33 au). **(D)** Bright image after dissection (NIRAF intensity: 4.77 au). T, thyroid; B, background; NIRAF, near-infrared autofluorescence.

Of the 51 PG images mapped by the expert, the novice judged 10 (19.6%) to be unconvincing as PGs (faint images) and 41 (80.4%) to be convincing (bright images) (**Table 1**). The NIRAF intensity of the faint images was weaker than that of the bright images (2.14 ± 0.48 vs. 3.38 ± 1.04 au, p = 0.001) (**Figures 2A, B**). In contrast, the NIRAF intensity of the bright group was sufficiently high (3.38 ± 1.04 au) that even the novice could be convinced that the bright images were PGs (**Figures 2C, D**). No difference in detectable depth was found between the bright and faint groups (p = 0.964) (**Table 1**). In a cross-tabulation analysis, there was no significant correlation of brightness with any of the other variables (**Table 2**).

**Table 2:** Correlations between brightness and other variables.

Variable	No. of PGs, n (%)	Brightness		p
		Faint, n (%)	Bright, n (%)	
Sex				0.667
Male	9 (17.6)	1 (10.0)	8 (19.5)	
Female	42 (82.4)	9 (90.0)	33 (80.5)	
Age				0.499
<52	25 (49.0)	6 (60.0)	19 (46.3)	
≥52	26 (51.0)	4 (40.0)	22 (53.7)	
Location of the PG				0.499
Superior	25 (49.0)	6 (60.0)	19 (46.3)	
Inferior	26 (51.0)	4 (40.0)	22 (53.7)	
Overlying tissue				0.291
Connective	26 (51.0)	7 (70.0)	19 (46.3)	
Fat	25 (49.0)	3 (30.0)	22 (53.7)	
BMI, kg/m^2^				1.000
Normal (18.5–23)	19 (37.3)	4 (40.0)	15 (36.6)	
Overweight/obese (≥23)	32 (62.7)	6 (60.0)	26 (63.4)	
Thyroiditis				1.000
Thyroiditis (-)	37 (72.6)	7 (70.0)	30 (73.2)	
Thyroiditis (+)	14 (27.4)	3 (30.0)	11 (26.8)	
Vitamin D, ng/mL				1.000
Low (<20)	16 (31.4)	3 (30.0)	13 (31.7)	
Normal (20–100)	35 (68.6)	7 (70.0)	28 (68.3)	

BMI, body mass index; PG, parathyroid gland.

## Discussion

Hypocalcemia, one of the most common complications of thyroid surgery, can be minimized by intraoperative localization of the PGs *via* NIRAF imaging, which is possible in 90–100% of cases (6–8, 11, 12, 17–20). Identification of unexposed PGs can prevent unnecessary manipulation of the PGs, damage to surrounding blood vessels, and inadvertent resection during surgery (6, 10). NIR light with a wavelength of 780 nm has different tissue penetration characteristics for different types of human tissue and is known to penetrate up to about 4 mm in human fat tissue (14). However, it was not previously known how deeply the autofluorescence emitted from the biological fluorophore of PGs can be detected by NIRAF imaging. To the best of our knowledge, this is the first report on a study measuring the depth at which unexposed PGs can be detected using NIRAF imaging during thyroidectomy.

The measured thickness of the overlying tissue in cases where the PG was identified through mapping was 1.23 mm on average, and the maximum thickness was 3.05 mm (**Table 1**). Although we measured the thickness between the surface of the adipose or connective tissue and the surface of the PG as the detectable depth, the detected autofluorescence is not produced by the surface of the PG, but by cells inside it. Considering this, it can be said that successful PG mapping *via* NIRAF imaging at depths of up to 3.05 mm almost matches the previously reported NIR light depth penetration of 4 mm (14). Early detection of PGs located 3 mm below the soft tissue, before the naked eye can detect them, can help surgeons to preserve parathyroid function. If the PG is not localized by NIRAF imaging, further dissection should be considered, with three possibilities (ectopic, previously resected, or deeply seated PG) (21, 22). We also investigated whether there was a difference between superior and inferior PGs regarding the type of overlying tissue (connective vs. adipose tissue). However, the results did not show any significant differences.

The NIRAF intensity of unexposed but mapped PGs increased by approximately 56% on average when they were exposed (p < 0.001). Additionally, there was a significant difference in the thickness of the overlying tissue of unexposed PGs according to tissue type (p < 0.001). PGs covered with connective tissue were often located at a shallow depth around the thyroid sheath, whereas PGs covered with fat tissue were often found deeply embedded in adipose tissue.

Stolik et al. found that 780 nm NIR light penetrates lipoma tissue, thyroid glands, and blood up to 4.04, 1.86, and 0.44 mm, respectively (14). Thus, although such events were not described or included in this study, we occasionally experience detection of superficially located intrathyroidal PGs by illumination of the thyroid surface with NIR light. In addition, since blood has a higher absorption coefficient than other tissues for light at NIR wavelengths (23, 24), the presence of blood vessels crossing the PG may reduce autofluorescence intensity due to absorption of the light by blood. Therefore, when the PG is not identified *via* the mapping process, it may be helpful to consider whether the PG is located beneath the vessel. In addition, PG mapping can be used to search for the PGs in the excised thyroid or central neck lymph node specimen if they are not detected during surgery.

Regardless of patient sex or age, location of the PG, overlying tissue type, patient BMI, thyroiditis, and serum vitamin D level (**Table 2**), the novice localized 41 of the 51 unexposed PGs (80.4%) that the expert was able to localize (**Table 1**). This means that even a novice can easily map unexposed PGs at a high rate of success. Thus, the use of PG mapping *via* NIRAF imaging would be expected to reduce the difficulties experienced by novices in identifying PGs during thyroidectomy.

In this study, the learning curve of PG mapping was not evaluated. Instead, we found that images considered bright by the novice had an average NIRAF intensity of 3.38 au (**Figure 2**). This value might serve as a reference point for novice practitioners in future studies. In order to more accurately evaluate the learning curve for interpretation of NIRAF images, further studies examining the cumulative sum learning curve will be necessary.

NIRAF imaging can assist surgeons in detecting PGs more easily, but it cannot provide information about parathyroid perfusion status. Many surgeons still rely on their experience to preserve function even after PG mapping. ICG angiography has been used as a method to assess the perfusion status of PGs at the end of surgery (25–27). Furthermore, intraoperative mapping angiography of the PGs (iMAP) has recently been introduced as a way to evaluate PG vascularity through intravenous ICG injection (28). Combining PG mapping, which can localize the parathyroid within 3 mm of the overlying tissue at the early stage of the surgery, with ICG angiography is expected to be useful in preserving PGs.

This study has several limitations, including the subjective nature of the measurements of detectable depth, which could potentially have affected the accuracy of the measurements. More objective methods should be considered in future studies. The small sample size and potential risk of false positive or negatives with NIRAF imaging are other limitations. It should also be noted that the imaging system utilized was still lab-built. Since there may be variations between instruments, additional verification of the detectable depth is necessary using other commercially available equipment.

In conclusion, NIRAF imaging can be used to map unexposed PGs at a maximum depth of 3.05 mm and an average depth of 1.23 mm. A novice was able to localize the PGs before they were visible to the naked eye at a high rate. These results can be used as reference data to aid location of the unexposed PG in thyroid surgery.

## Data availability statement

The raw data supporting the conclusions of this article will be made available by the authors, without undue reservation.

## Ethics statement

The studies involving human participants were reviewed and approved by Institutional Review Board (IRB) of Kosin University Gospel Hospital (IRB No. 2021-07-008-001). The patients/participants provided their written informed consent to participate in this study.

## Author contributions

All authors participated in the study design and concept, collected and analyzed data, played a role in writing and reviewing the article to ensure it was of high quality, and approved the final version to be published. Conceptualization: YH, HL, Y-CA, KL Design and methodology: YH, HL, YJK, YKK, Y-CA, KL Conduction of the study: YH, YKK, Y-CA, KL Statistical analysis and interpretation: YH, YKK, Y-CA, KL Writing—original draft preparation: YH Writing—review and editing: YH, HL, YJK, YKK, Y-CA, KL Resources: KL Supervision: HL, YKK, Y-CA, KL.
